# Empathy in mild cognitive impairment: a preliminary clinical comparative study in Southern Switzerland using the interpersonal reactivity index (IRI) and the story-based empathy task (SET)

**DOI:** 10.3389/fnagi.2025.1661172

**Published:** 2025-09-09

**Authors:** L. Arnaud, L. Morellini, L. G. Rege-Colet, A. Pagnamenta, E. Fontana, M. Lissi, R. Morese, L. Sacco

**Affiliations:** ^1^Department of Neurology, Neuropsychology and Speech Therapy Unit, Neurocenter of Southern Switzerland, Ente Ospedaliero Cantonale, Lugano, Switzerland; ^2^Clinique romande de réadaptation, Sion, Switzerland; ^3^Clinical Trial Unit, Ente Ospedaliero Cantonale, Lugano, Switzerland; ^4^Departement of Intensive Care Medicine, Ente Ospedaliero Cantonale, Lugano, Switzerland; ^5^Division of Pneumology, University of Geneva, Geneva, Switzerland; ^6^Faculty of Biomedical Sciences, Università della Svizzera Italiana, Lugano, Switzerland; ^7^Institute of Communication and Public Policy (ICPP), Faculty of Communication, Culture and Society, Università della Svizzera italiana, Lugano, Switzerland

**Keywords:** mild cognitive impairment, social cognition, empathy, theory of mind, interpersonal reactivity index, story-based empathy task

## Abstract

**Introduction:**

Mild Cognitive Impairment (MCI) is a transitional stage between normal aging and dementia, frequently associated with subtle deficits in social cognition. Empathy, a core component of social cognition, encompasses both affective and cognitive dimensions and may be compromised even in prodromal phases of neurodegenerative conditions. Despite its clinical relevance, empathy in MCI remains underexplored, and standardized assessment tools are seldom used in routine diagnostics. Theory of mind is another important aspect of social cognition, and, as with empathy, it is unclear how it is affected at the MCI stage. This study aimed to investigate empathy and theory of mind abilities in individuals with MCI, excluding cases attributable to prodromal frontotemporal dementia, using two validated instruments: the Interpersonal reactivity index (IRI) and the Story-based empathy task (SET).

**Methods:**

We conducted a case–control study involving 23 individuals with MCI and 25 cognitively healthy controls. All participants completed the IRI, while a subsample (19 MCI patients) also underwent the SET. The clinical group included both amnestic and non-amnestic MCI subtypes, with heterogeneous etiologies. Group comparisons were performed on IRI subscales and SET indices to assess both self-reported empathy traits and performance-based socio-cognitive abilities.

**Results:**

The MCI group exhibited significantly lower scores in the IRI subscales of Empathic Concern and Perspective Taking compared to controls, indicating concurrent affective and cognitive empathy impairments. While no significant differences emerged in individual SET subcomponents, the MCI group showed a significantly lower global SET score, suggesting reduced integrative socio-cognitive performance. These findings should be interpreted with caution given the limited sample size and clinical heterogeneity, which make this a preliminary study.

**Conclusion:**

These findings provide preliminary evidence of early empathy-related and theory of mind alterations in MCI, supporting the inclusion of social cognition assessments in standard neuropsychological protocols. The combined use of self-report and task-based instruments may enhance early identification of socio-emotional dysfunctions and inform personalized clinical interventions. The preliminary nature of this study is mainly due to the small sample size and the heterogeneous clinical profiles, which limit generalizability but highlight the need for replication in larger cohorts.

## 1 Introduction

Mild Cognitive Impairment (MCI) is, by definition, considered a prodromal stage of dementia, representing a transitional phase between healthy aging and neurodegenerative decline (Anderson, 2019; Breton et al., 2019). In this phase, patients show an increased risk of developing dementia in the following years, although their daily functioning remains preserved (Petersen, 1996; Smith et al., 1996). The term MCI was first defined by Petersen et al. (1997) to describe this intermediate state, in which patients do not yet fulfill diagnostic criteria for dementia (Albert et al., 2011).

Petersen (2016) proposed a distinction between amnestic (aMCI) and non-amnestic (naMCI) subtypes. Respectively, aMCI is primarily associated with episodic memory impairments, while naMCI involves other cognitive domains, including executive functions, language, or visuospatial abilities, and is typically considered prodromal to Alzheimer’s disease (Albert et al., 2011). Importantly, MCI represents a heterogeneous clinical condition, and its underlying etiology can differ substantially. Among the primary etiological subtypes are MCI associated with Alzheimer’s disease (AD-MCI), Parkinson’s disease (PD-MCI), frontotemporal dementia (FTD-MCI), cerebrovascular pathology (vascular MCI; VaMCI), and Lewy body disease (LB-MCI) (Benussi et al., 2021; McKeith et al., 2020; Litvan et al., 2011). The DSM-5-TR describes this condition under the term “mild neurocognitive disorder,” emphasizing a mild cognitive decline, in one or more cognitive domains, that does not interfere with independence in daily functioning and is not explained by delirium or psychiatric disorders (American Psychiatric Association, 2022).

The cognitive domains potentially affected in MCI include memory, attention, executive functioning, language, perceptual-motor and social cognition. In particular, social cognition refers to the cognitive processes involved in understanding, reacting to, and interacting with others (Frith and Blakemore, 2006). It comprises four main subdomains: empathy, theory of mind (ToM), emotion recognition, and social decision-making (Decety and Jackson, 2004; Shamay-Tsoory, 2010; Lamm et al., 2011; Premack and Woodruff, 1978). Despite being recognized in the DSM-5 as a key cognitive domain, social cognition is still rarely assessed in clinical neuropsychological practice (Cerami et al., 2025). A recent international survey confirmed this gap, pointing to the lack of standardized tools, training, and guidelines as major barriers in memory clinics (Cerami et al., 2025). Within this domain, empathy represents a core construct encompassing both affective and cognitive components (Decety and Jackson, 2004; Zaki and Ochsner, 2012). Affective empathy refers to automatic processes such as imitation and emotional contagion, whereas cognitive empathy involves understanding and mentalizing others’ emotional states, overlapping with Theory of Mind abilities (Premack and Woodruff, 1978; Singer, 2009; Moore et al., 2015). To our knowledge, this study is the first to combine a self-report measure of empathy (IRI) with a performance-based task (SET) in a population with MCI, providing a novel and multidimensional approach to the evaluation of socio-cognitive abilities in this condition. One of the most widely used and validated instruments for assessing empathy in both clinical and non-clinical populations is the Interpersonal reactivity index (IRI; Davis, 1980, 1983). It enables the distinction between cognitive and affective components of empathy through four subscales: Perspective Taking (PT), Fantasy (F), Empathic Concern (EC), and Personal Distress (PD). Specifically, the PT subscale reflects cognitive empathy—defined as the capacity to adopt another individual’s psychological perspective—while the EC subscale captures affective empathy, namely the tendency to feel warmth, compassion, and concern for others in distress (Davis, 1980, 1983; Giacomucci et al., 2022; Quaranta et al., 2023).

Another instrument used to assess social cognition is the Story-based empathy task (SET), a non-verbal tool developed to evaluate both affective and cognitive components of *Theory of Mind* (Dodich et al., 2015). The SET is based on short comic strips and comprises two experimental conditions—intention attribution (SET-IA) and emotion attribution (SET-EA)—as well as a control condition assessing physical causality inference (SET-CI). Each trial presents participants with an incomplete comic strip consisting of an upper row (the story) and three possible endings, from which they must select the most appropriate one. Correct responses are scored with one point, and the maximum score for each condition is 6, with a total maximum score of 18. Prior to making their choice, participants are asked to verbally describe the story and predict a plausible ending to ensure comprehension of the task. The SET has been validated on the Italian population, where performance has been shown to be influenced by age and education level, but not by gender (Dodich et al., 2015). Compared to other social cognition tasks, the SET is brief (15–20 min), easy to administer, and specifically designed to capture subtle socio-cognitive impairments even in early neurodegenerative conditions. It was chosen as a screening test in an intentional battery for MCI (Boccardi et al., 2022). It has shown particular utility in detecting deficits in behavioral variant Frontotemporal Dementia (bvFTD) and Amyotrophic Lateral Sclerosis (ALS), where early impairments in emotion attribution correlate with grey matter reduction in fronto-temporal and limbic structures (Dodich et al., 2015; Adenzato and Poletti, 2013). Standardized and ecologically valid instruments such as the SET may constitute a valuable addition to routine neuropsychological assessments, particularly in clinical populations where social cognition is often underexplored—including Parkinson’s disease, Huntington’s disease, brain tumors, and traumatic brain injury—and may be especially useful in the assessment of patients with MCI, in whom subtle empathy disturbances could represent early markers of neurodegenerative progression (Boccardi et al., 2022; Dodich et al., 2015; Campanella et al., 2014; McDonald and Flanagan, 2004).

The distinction between cognitive and affective components of empathy is not only supported by behavioral evidence, but also by converging findings from neuroimaging studies that have identified distinct neural substrates underlying each dimension. According to the model proposed by Shamay-Tsoory (2010), affective empathy is primarily associated with activity in the inferior frontal gyrus (IFG), anterior insula, anterior cingulate cortex (ACC), and inferior parietal lobule—regions implicated in emotional processing and interoception. In contrast, cognitive empathy engages a network involving the medial prefrontal cortex (mPFC), temporoparietal junction (TPJ), superior temporal sulcus (STS), and temporal poles, all of which are known to support higher-order social cognition and mentalizing processes (Shamay-Tsoory, 2010).

Emerging literature from 2022 to 2024 has deepened our understanding of empathy alterations in MCI. Quaranta et al. (2024) reported that patients with AD-related MCI displayed significantly reduced Perspective Taking and Fantasy scores on the IRI, while paradoxically showing increased Empathic Concern—suggesting an imbalance between cognitive and affective empathy. Interestingly, these changes were not explained by executive dysfunction alone, supporting the hypothesis of early disruption in specific brain networks regulating empathy. MRI data confirmed a correlation between IRI scores and structural alterations in regions including the TPJ and amygdala, indicating a disease-specific neural basis rather than a generic cognitive decline (Quaranta et al., 2024).

Complementing these findings, Giacomucci et al. (2022) used FDG-PET to demonstrate that impairments in cognitive empathy, meaning reduced Perspective Taking scores on the IRI, correlated with hypometabolism in the right middle frontal gyrus (a region associated with the dorsolateral prefrontal cortex), both in MCI and AD. Moreover, increased Personal Distress—a proxy for emotional contagion—was observed along the continuum from Subjective cognitive decline (SCD) to AD, suggesting that affective dysregulation may begin in the prodromal stage and might even precede cognitive decline. These authors emphasize the dissociation between affective and cognitive empathy in early AD pathology, potentially related to differential involvement of mirror neuron systems and emotion regulation regions such as the superior parietal lobule and superior temporal gyrus.

Despite the growing body of neuroimaging research elucidating the neural substrates of empathy, no studies to date have examined brain correlates associated with performance on the SET. As a result, there is currently no available evidence linking SET outcomes to structural or functional brain alterations, limiting our understanding of the neurobiological mechanisms underlying socio-cognitive abilities as measured by this instrument. Furthermore, empirical data on the use and validity of the SET in detecting social cognition impairments specifically in individuals with MCI are currently lacking, underscoring the need for further research in this population.

Although neuroimaging evidence has clarified the neural substrates underlying cognitive and affective empathy, empirical findings regarding social cognition—particularly empathy—in individuals with MCI remain inconsistent. Several studies have attempted to characterize empathic dysfunctions in MCI, yet the results are heterogeneous and often contradictory. This variability has been attributed to methodological limitations such as small sample sizes, differences in diagnostic criteria, and the lack of standardized assessment protocols (Sacco et al., 2023a,b). While some investigations have reported significant impairments in both cognitive and affective components of empathy among MCI patients, others have failed to detect marked deviations from healthy controls, particularly in affective subscales such as Personal Distress (Quaranta et al., 2024).

Recent findings suggest that alterations in empathy-related brain regions may be observable even in preclinical populations and could represent early biomarkers of neurodegenerative processes (Giacomucci et al., 2022). In this broader context, social cognition deficits have been consistently described across various neurodegenerative conditions, including Alzheimer’s disease and Parkinson’s disease (Kessels et al., 2021; Rossetto et al., 2018; Adenzato et al., 2019), as well as in prodromal stages such as MCI. However, studies specifically focused on empathy in MCI remain scarce. Therefore, the overall picture remains fragmented, and further research using rigorous, standardized methodologies is needed to delineate the profile of social cognition and empathy in MCI more conclusively (Morellini et al., 2022a,b; Arioli et al., 2018; Bora and Pantelis, 2016).

In light of these considerations, the aim of our study is to investigate affective and cognitive empathy, as well as theory of mind, in patients with mild cognitive impairment (MCI) not due to prodromal FTD, using reliable and feasible tests such as the IRI and the SET. We compared their performance with that of healthy controls. More broadly, we aim to determine whether alterations in empathy may represent a reliable clinical marker for the early identification of dementia not related to fronto-temporal degeneration.

## 2 Materials and methods

### 2.1 Participants

We conducted a case–control study involving a clinical group of individuals with MCI (*N* = 23) and a control group of cognitively healthy older adults (*N* = 25). While all participants completed the full assessment protocol, four individuals from the clinical group were excluded specifically from the analyses involving the Story-based empathy task (SET). The exclusion was necessary because these participants were unable to independently comprehend the task instructions and/or interpret the narrative content, leading to unreliable performance. Consequently, SET-related analyses were conducted on a subsample of 19 MCI participants.

**Figure 1:**
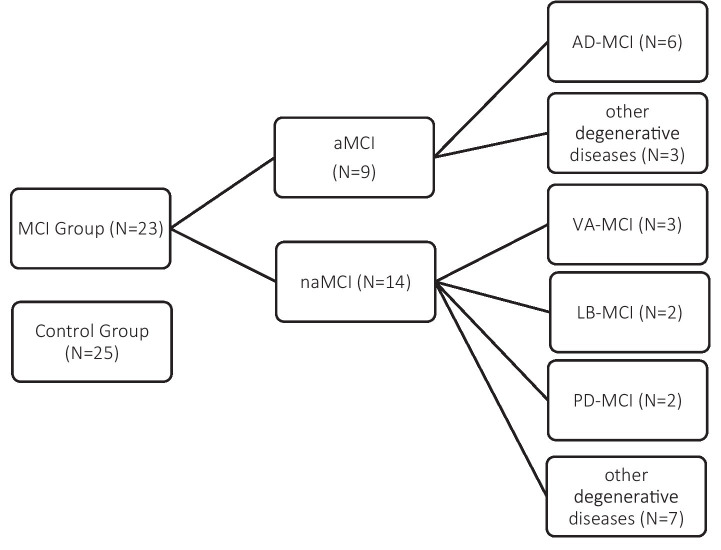
Composition of the study sample. The MCI group (*N* = 23) was subdivided into amnestic (aMCI; *N* = 9) and non-amnestic (naMCI; *N* = 14) subtypes, with further classification based on etiology. The control group included 25 cognitively healthy participants.

The final clinical sample (*N* = 23) included amnestic MCI (aMCI; *N* = 9)—of whom 6 were diagnosed with Alzheimer’s disease-related MCI (AD-MCI) and 3 with other degenerative conditions—and non-amnestic MCI (naMCI; *N* = 14), with heterogeneous etiologies:

Parkinson’s disease (PD-MCI, *N* = 2), Lewy body disease (LB-MCI, *N* = 2), vascular dementia (VaMCI, *N* = 3), and other medical conditions (*N* = 7) (Petersen, 1996; Albert et al., 2011; Benussi et al., 2021; McKeith et al., 2020; Litvan et al., 2011; American Psychiatric Association, 2022) (see Figure 1). Following the exclusion of four clinical participants for the reasons previously described, the SET-related analyses were conducted on a clinical sample comprising 19 individuals. The four excluded participants belonged to the following diagnostic subgroups: one individual with AD-MCI, one with LB-MCI, and two with VaMCI. Patients in the clinical group were consecutively recruited, upon obtaining informed consent, during routine neuropsychological assessments at the Memory Clinic of the Ente Ospedaliero Cantonale (EOC). The control group was recruited through the Alzheimer Ticino Association, which supported the identification of healthy older adults and provided the informed consent to participate in the study.

Inclusion criteria for the clinical group comprised: (1) a diagnosis of MCI with the exception of prodromal Fronto temporal Dementia (Petersen, 1996; Albert et al., 2011; Benussi et al., 2021; McKeith et al., 2020; Litvan et al., 2011; American Psychiatric Association, 2022); (2) preserved capacity for discernment and comprehension sufficient to provide informed consent and complete the study procedures; (3) a score below 26 on the Montreal Cognitive Assessment (MoCA); and (4) age greater than 60 years.

Exclusion criteria included: (1) current or past diagnosis of major depressive disorder or other relevant psychiatric conditions that could interfere with cognitive performance or study participation.

Participants in the control group met the following inclusion criteria: (1) absence of neurological or psychiatric diagnoses, confirmed through clinical interview; (2) MoCA score above 26, indicating normal cognitive functioning; and (3) age over 60 years.

Exclusion criteria were identical to those applied to the clinical group, specifically the presence of major depression or other related psychiatric disorders.

### 2.2 Procedure

All participants, both in the clinical and control groups, underwent a standardized assessment protocol after providing written informed consent. Demographic variables—including age, gender, and years of education—were collected prior to testing.

To assess global cognitive functioning and confirm eligibility criteria, all participants completed the Montreal Cognitive Assessment (MoCA) (Nasreddine et al., 2019), a widely used cognitive screening tool designed to detect mild cognitive deficits.

To evaluate empathy, we administered the IRI (Davis, 1980, 1983), a self-report questionnaire that captures both cognitive and affective components of empathy. It comprises four subscales: *Fantasy* (F) and *Perspective Taking* (PT) assess cognitive empathy, while *Empathic Concern* (EC) and *Personal Distress* (PD) evaluate affective empathy. Specifically, the F subscale reflects the tendency to identify with fictional characters and situations; PT measures the ability to adopt others’ psychological perspectives; EC captures feelings of compassion toward others in distress; and PD assesses self-oriented discomfort in response to others’ suffering.

In addition to the IRI, participants completed the SET (Dodich et al., 2015), a non-verbal instrument developed to assess social cognitive abilities, particularly the attribution of intentions and emotional states. The task is based on brief comic strips and includes two experimental conditions—*Intention Attribution* and *Emotion Attribution*—as well as a control condition involving physical causality. For each item, participants are required to infer the most plausible ending among three alternatives and justify their choice by first describing the story. This task allows for an ecologically valid assessment of Theory of Mind skills through visual narratives, and is particularly sensitive to early socio-cognitive impairments. The total score ranges from 0 to 18, with higher scores reflecting better performance. Its ease of administration (15–20 min) and validation in the Italian population make it a suitable tool for both clinical and research contexts (Dodich et al., 2015; Adenzato and Poletti, 2013).

## 3 Statistical analysis

Descriptive statistics were computed for all variables. Categorical variables were presented as absolute frequencies and percentages. For continuous variables, data were reported as means and standard deviations (*M* ± SD) when normally distributed, or as medians with 25th – 75th percentile when non-normal distributions were observed. Group comparisons were conducted using the chi-square test (*χ*^2^) for categorical variables, the independent-samples *t* test for normally distributed continuous variables, and the Mann–Whitney *U* test for non-normally distributed continuous variables. All analyses were performed using *Stata* (Version 17.0; StataCorp LLC, College Station, TX, USA). Statistical significance was set at *p* < 0.05 for all comparisons.

## 4 Results

The final sample included 23 participants in the MCI group and 25 cognitively healthy individuals in the control group. No significant differences were observed between groups with respect to sex distribution (*p* = 0.154), or education level (*p* = 0.547). Similarly, age did not differ significantly between groups (*p* = 0.445), with a median age of 71 years in the MCI group and 69 years in the control group (see Table 1).

**Table 1:** Demographic characteristics of the clinical and control groups.

	Category	MCI Group (*n* = 23)	Control Group (*n* = 25)	*p*-value
Education level (%)	Low (5–9 yrs)	7 (30.4%)	11 (44.0%)	
	Medium (9–13 yrs)	10 (43.5%)	10 (40.0%)	
	High (>13 yrs)	6 (26.1%)	4 (16.0%)	
Total	23 (100%)	25 (100%)	0.547
Sex (%)	Male	16 (69.6%)	12 (48.0%)	
	Female	7 (30.4%)	13 (52.0%)	
	Total	23 (100%)	25 (100%)	0.154
Age (years)	Median (25th-75th)	71 (68–76)	69 (66–76)	
				0.445

Frequencies and percentages are reported for categorical variables (education level, sex). Median score and the 25th and 75th percentiles, are reported for age. No statistically significant group differences were observed.

Median scores and the 25th and 75th percentiles for the IRI subscales and total scores are presented in Table 2. Statistically significant group differences emerged in two subscales. The MCI group showed significantly lower scores in Empathic Concern (EC; *median* = 21, *25th–75th* = 18–24) compared to controls (*median* = 23, *25th–75th* = 21–25; *p* = 0.019), as well as in Perspective Taking (PT; *median* = 14*, 25th–75th* = 12–18 vs. *median* = 17*, 25th–75th* = 16–19; *p* = 0.041). No significant differences were found for the Personal Distress (PD) or Fantasy (F) subscales (*p* = 0.296 and *p* = 0.541, respectively), nor for the total IRI score (*p* = 0.091), although a trend toward lower total empathy scores in the MCI group was observed.

**Table 2:** Interpersonal reactivity index (IRI) scores in clinical and control groups.

	MCI group (*n* = 23)	Control group (*n* = 25)	*p*-value
IRI_PD	12 (8–14)	9 (6–13)	0.296
IRI_EC	21 (18–24)	23 (21–25)	0.019*
IRI_F	14 (10–17)	14 (12–17)	0.541
IRI_PT	14 (12–18)	17 (16–19)	0.041*
IRI_TOT	60 (52–67)	63 (60–68)	0.091

Median scores and the 25th and 75th percentiles (in parentheses) are reported for each IRI subscale: Personal Distress (PD), Empathic Concern (EC), Fantasy (F), Perspective Taking (PT), and the total score (TOT). Patients with MCI showed significantly lower Empathic Concern and Perspective Taking compared to controls (*p* < 0.05), indicating concurrent impairments in both affective and cognitive empathy components.

The SET was completed by 19 individuals in the MCI group and all 25 participants in the control group. As shown in Table 3, no statistically significant differences were observed between groups in the Intention Attribution (SET_IA), Emotion Attribution (SET_EA), or Causal Inference (SET_CI) conditions (*p* = 0.230, 0.173, and 0.668, respectively). However, a significant group difference emerged in the Global Score (SET_GS), with lower overall performance in the MCI group (*median* = 16, *25th–75th* = 15–17) compared to controls (*median* = 17, *25th–75th* = 16–18; *p* = 0.027), suggesting subtle impairments in integrated social cognitive abilities among individuals with MCI.

**Table 3:** Story-based empathy task (SET) scores in clinical and control groups.

	MCI Group (*n* = 19)	Control Group (*n* = 25)	*p*-value
SET_IA	5 (5–6)	6 (5–6)	0.230
SET_CI	5 (5–6)	6 (5–6)	0.668
SET_EA	6 (5–6)	6 (6–6)	0.173
SET_GS	16 (15–17)	17 (16–18)	0.027*

Median scores and the 25th and 75th percentiles (in parentheses) are reported for each condition: Intention Attribution (IA), Causal Inference (CI), Emotion Attribution (EA), and the Global Score (GS). Although no significant differences were found in individual subcomponents, the MCI group obtained a significantly lower Global Score (*p* < 0.05), reflecting subtle deficits in integrated socio-cognitive processing compared to controls.

## 5 Discussion

This study aimed to investigate empathy and social cognition in individuals with MCI using two complementary instruments: the Interpersonal reactivity index (IRI) and the Story-based empathy task (SET). The findings reveal subtle but significant differences between the MCI and control groups in specific components of empathy and social cognition, offering valuable insights into the early socio-cognitive changes associated with neurodegenerative processes not related to the prodromal stage of frontotemporal dementia.

With regard to empathy, as measured by the Interpersonal reactivity index (IRI), our MCI group demonstrated significantly lower scores in both the Empathic Concern (EC) and Perspective Taking (PT) subscales compared to healthy controls. These subscales reflect, respectively, the affective and cognitive components of empathy (Davis, 1980, 1983), and their concurrent reduction suggests an early-stage decline in both emotional resonance and the ability to adopt another’s psychological perspective. This pattern stands in contrast to several recent studies which have reported a dissociation between cognitive and affective empathy in MCI, typically characterized by reduced Perspective Taking and preserved or even increased Empathic Concern (Giacomucci et al., 2022; Quaranta et al., 2024). Those findings have been interpreted as an early breakdown of cognitive empathy mechanisms, possibly due to executive dysfunction (Sacco et al., 2023a,b), with affective empathy being more resilient or even amplified in response to declining social awareness. In contrast, our results indicate a concomitant decline in both components, suggesting a more global deterioration of empathic processing in our MCI sample. This may reflect a broader neurodegenerative impact on the neural circuits subserving empathy, or be related to methodological differences, such as the use of self-report versus informant-report measures, or sample heterogeneity in terms of MCI etiology.

Regarding Theory of Mind, the results obtained from the SET revealed no significant group differences in individual subcomponents (i.e., Intention Attribution, Emotion Attribution, and Causal Inference), but the Global Score was significantly lower in the MCI group. This finding suggests a subtle but measurable reduction in overall socio-cognitive integration, consistent with prior studies highlighting the SET’s sensitivity in detecting early deficits in clinical populations (Dodich et al., 2015; Adenzato and Poletti, 2013). While existing neuroimaging research has explored the neural correlates of IRI performance (Quaranta et al., 2024; Giacomucci et al., 2022), to date, no studies have investigated the brain correlates of SET performance. Furthermore, there is a lack of empirical validation for the SET in the context of MCI, limiting our understanding of its utility for detecting socio-cognitive alterations in this population. This gap aligns with broader observations from the SIGNATURE initiative, which highlights the urgent need for validated, cross-culturally applicable tools for the assessment of social cognition in neurocognitive disorders (Cerami et al., 2025).

Taken together, our results contribute to the growing literature on empathy and social cognition in MCI by identifying early signs of disruption in both affective and cognitive components of empathy, as well as in integrated socio-cognitive functioning. These alterations, although subtle, may serve as potential clinical markers of neurodegenerative progression and underscore the importance of including an evaluation of socio-emotional abilities in the routine assessment of patients with cognitive complaints (Kessels et al., 2021; Bora and Pantelis, 2016). From a clinical perspective, our results highlight the potential value of systematically including empathy and theory of mind assessments in neuropsychological protocols for MCI. Self-report instruments such as the IRI may help identify subjective difficulties in affective and cognitive empathy, while performance-based measures like the SET can reveal subtle impairments in socio-cognitive integration. Combined use of these tools may therefore enhance early detection of at-risk individuals and inform tailored interventions, for example cognitive-behavioral strategies, caregiver training, or programs aimed at preserving social engagement. Moreover, our findings align with recent research demonstrating the strong interdependence between executive functions and theory of mind in MCI (Clemente et al., 2023). This suggests that socio-cognitive decline may be partly mediated by executive dysfunction, underscoring the importance of assessing and addressing both domains in order to achieve a comprehensive understanding of patients’ social functioning.

These findings are consistent with recent international data showing that, despite the recognized clinical importance of social cognition, its systematic assessment remains largely absent in memory clinics. Real-life constraints—such as limited time, lack of training, and the absence of harmonized guidelines—contribute to the underutilization of socio-cognitive tools in clinical settings (Cerami et al., 2025). Further investigation using neuroimaging methods is warranted to clarify whether these empathy impairments stem from specific regional brain alterations, or reflect a more general decline in cognitive and emotional integration capacities. However, several limitations must be acknowledged. First, the heterogeneity of the clinical group, which included individuals with different MCI subtypes and etiologies (e.g., aMCI vs. naMCI), may have introduced variability that could obscure more specific socio-cognitive profiles. The exclusion of frontotemporal dementia makes this limitation less impactful. Although our sample size did not allow for stratified analyses, future studies should address whether empathy and theory of mind deficits follow distinct patterns across MCI subtypes, as this could improve the specificity of clinical markers. Second, the sample size was relatively small and regionally confined (Canton Ticino), thus limiting the generalizability of the findings. Third, although no significant differences were observed in education levels between groups, the potential influence of educational attainment on empathy and social cognition cannot be excluded, as previous research has shown an association between higher education and better performance on mentalizing tasks (Maddaluno et al., 2022). Another important methodological aspect concerns the statistical power of the analyses. Because this was an exploratory study, no *a priori* sample size calculation was performed, which increases the likelihood of type II errors. A post-hoc power analysis on the main outcome variable (IRI total score) yielded an estimated power of 0.473 (95% CI: 0.466–0.479). This result confirms that the study was underpowered relative to the conventional 0.80 threshold and reinforces the need for replication in larger cohorts to reliably detect subtle socio-cognitive differences between groups.

A further factor potentially influencing our results is the role of executive functioning and sex differences in empathy and theory of mind. Previous research has demonstrated that executive functions play a moderating role in emotion recognition and perspective-taking among older adults, and that these effects may differ by sex (Thompson and Voyer, 2014; Pua and Yu, 2024). Women, for example, often show an advantage over men in both emotion recognition and empathic sensitivity. Although our sample size did not allow for stratified analyses, we cannot exclude that part of the variability in empathy measures may reflect sex-related differences or the contribution of executive dysfunction. Future studies with larger samples should therefore consider sex and executive functioning as potential moderators of socio-cognitive decline in MCI. Future studies should aim to replicate these findings in larger, more homogeneous samples, and further explore the ecological and diagnostic validity of instruments such as the SET in MCI populations as screening test. The integration of neuroimaging techniques could help elucidate the neural substrates underlying SET performance and refine our understanding of socio-cognitive changes in prodromal neurodegenerative conditions. Moreover, the inclusion of caregiver reports or semi-structured clinical interviews could provide complementary information about real-life social functioning, which is not always captured through standardized tasks. From a clinical perspective, early identification of empathic or social cognitive alterations may inform intervention strategies such as cognitive-behavioral therapy, social skills training, or caregiver education programs. Such approaches may help maintain social functioning and quality of life in individuals at risk of developing dementia.

In conclusion, this study provides preliminary but meaningful evidence that specific components of empathy and theory of mind are already affected in the early stages of cognitive impairment. These findings support the relevance of combining self-report and performance-based measures to detect subtle socio-emotional changes, and highlight the need for more comprehensive and multidimensional approaches to the assessment and treatment of MCI. Moreover, as emphasized by recent harmonization efforts, the adoption of brief, clinically feasible, and ecologically valid instruments is essential for bridging the gap between research and routine practice in the assessment of socio-cognitive domains (Cerami et al., 2025).

## 6 Conclusion

This study sheds light on the early socio-cognitive alterations in individuals with MCI, revealing specific impairments in both affective and cognitive empathy, as well as subtle deficits in integrated theory of mind abilities. By combining self-report and performance-based instruments, we underscore the clinical value of multidimensional empathy assessment in this population. Despite its preliminary nature, this study is valuable as a first step in addressing an under-explored domain in MCI. It helps identify the most sensitive tools for detecting early socio-cognitive alterations. Furthermore, it highlights the importance of larger, stratified samples, integration with neuroimaging and the inclusion of caregiver perspectives, thereby guiding the design of future work. In this sense, the present preliminary findings contribute towards more rigorous research in this emerging area of clinical neuroscience.

## Data Availability

The raw data supporting the conclusions of this article will be made available by the authors, without undue reservation.
